# Prevalence of self-reported hypercholesterolaemia and its relation to dietary habits, in Greek adults; a national nutrition & health survey

**DOI:** 10.1186/1476-511X-5-5

**Published:** 2006-03-12

**Authors:** George A Milias, Demosthenes B Panagiotakos, Christos Pitsavos, Dimitra Xenaki, George Panagopoulos, Christodoulos Stefanadis

**Affiliations:** 1 Department of Nutrition – Dietetics, Harokopio University, Athens, Greece; 2 First Cardiology Clinic, School of Medicine, University of Athens, Greece; 3 Unilever Institute, Athens, Greece

## Abstract

**Background:**

The strong causal role of hypercholesterolaemia on the progression of atherosclerosis and subsequently on the development of cardiovascular disease is well described. Main aim of this study was to evaluate the prevalence of self-reported hypercholesterolaemia and its relation to nutritional habits, in a representative nationwide sample of adult Greek population.

**Methods:**

Cross sectional survey. Based on a multistage sampling, 5003 adults (18 – 74 yr) were enrolled (men: 48.8%, women: 51.2%). All participants were interviewed by trained personnel who used a standard questionnaire. The questionnaire included demographic and socioeconomic characteristics, medical history, lifestyle habits and nutritional assessment.

**Results:**

The prevalence of self-reported hypercholesterolaemia was 16.4% in men and 21.8% in women (P < 0.001). Hypercholesterolaemic status was positively associated with the prevalence of hypertension, diabetes mellitus, renal failure, obesity and physical inactivity, and inversely with the prevalence of smoking. The analysis of reported food consumption patterns showed that consumption of fish, fruits and juices, cereals, and low fat milk and yogurt was significantly higher among hypercholesterolaemic participants while the opposite was observed for food items as red meat, pork, egg, full fat dairy products and desserts.

**Conclusion:**

Hypercholesterolaemia seems to affect a large part of Greek population. It is hopeful that hypercholesterolaemics may have started adopting some more healthy nutritional behaviour compared to normocholesterolaemic ones.

## Background

Cardiovascular disease is the leading cause of death worldwide affecting millions of people in both developed and developing countries. In particular, in most European countries cardiovascular disease contributes about 40% to all cause mortality [1]. Accumulating evidence coming up from numerous studies [2-6], have revealed the strong causal role of hypercholesterolaemia on the progression of atherosclerosis and subsequently on the development of cardiovascular disease. According to the World Health Organization (WHO) it is estimated that high cholesterol causes around a third of all cardiovascular disease worldwide, and that 105 million people in the USA have cholesterol levels that are a cardiovascular risk [7].

It should be noted that the strength of the relationship between hypercholesterolaemia and atherosclerosis might be influenced by several lifestyle related factors, like smoking, physical activity and psychosocial conditions [2]. It is also known that well balanced dietary patterns, like the Mediterranean diet, are strongly related with blood lipids levels, as well as with the prevalence and the management of dyslipidemia [8-12]. Furthermore, it is encouraging that, primary and secondary prevention trials [13-15] have provided strong evidence that lowering serum cholesterol levels can be translated into a reduction in mortality rates from cardiovascular disease.

The limited current data regarding the prevalence of hypercholesterolaemia in Greece, in combination with the considerable variability of hypercholesterolaemia between countries, as well as within countries, and between different areas and population groups [16], motivated us to conduct a cross-sectional study in a nationwide sample of Greek adults. The study was also undertaken with aim to evaluate the association between nutritional habits and the prevalence of hypercholesterolaemia.

## Methods

### Study sample

In the present study, which was carried out in Greece in autumn of 2004, 5003 adults (18 – 74 years) participated. In order to be the study's sample as more representative as possible, the selection of the participants (men: 48.8%, women: 51.2%) was random, multistage, nationwide (including 66.6% urban and 33.4% rural areas) and it was based on the age-gender distribution of Greek population provided by the National Statistical Service according to the census of 2000 (Table 1). Furthermore, the sampling anticipated enrolling only one participant per household. All participants were interviewed with telephone by trained personnel (cardiologists, dieticians and nurses) who used a standard questionnaire.

**Table 1:** Age - gender distribution of the study's population and the Greek population.

	**Study's Population**		**Greek Population**	
**Age (years)**	Men	Women	Men	Women

18–24	11.2 %	10.0 %	13.7 %	12.4 %
25–34	22.5 %	21.0 %	21.2 %	19.9 %
35–44	20.0 %	19.7 %	19.4 %	19.3 %
45–54	17.8 %	17.7 %	16.9 %	16.9 %
55–64	14.6 %	15.8 %	14.7 %	15.8 %
65–74	13.8 %	15.8 %	14.1 %	15.7 %

### Investigated measurements

The questionnaire addressed especially: a) individual demographic and socioeconomic characteristics (age, gender, schooling, occupation, residence); b) information relevant to health status assessment (hypercholesterolaemia, hypertension, diabetes mellitus and renal failure); c) food consumption pattern; d) lifestyle habits, such as smoking status and level of physical activity; and e) characteristics related to body composition (recalled weight and height).

Regarding the assessment of hypercholesterolaemic status, the subjects were considered as hypercholesterolaemic if they were reporting that they had been previously diagnosed by a registered physician (type of answer: yes/no). In case of positive answer, they were asked to report the most recent measurement of total cholesterol in serum, and to provide data relevant to the selected way of treatment (drug treatment or dietary treatment or drug and dietary treatment or no treatment). Respective information (prior diagnosis, intensity of problem and selected way of treatment) concerning hypertension, diabetes mellitus and renal failure were retrieved too. Family history of all the aforementioned health problems was also recorded (type of answer: yes /no / don't know for father, mother, brother and sister).

Relatively to the nutritional component of the questionnaire, the participants were asked to report their average weekly consumption (in servings) of a list of food items of all different food groups. The educational level of the participants (as a proxy of social status) was measured by the years of schooling (Group I: < 9 years, Group II: up to high school or technical colleges (10 – 14 years) and Group III: university). Current smokers were defined as those who smoked at least one cigarette per day; never smokers those who have never tried a cigarette in their life and former smokers were defined as those who had stopped smoking for at least one year. Physically active were considered all the participants who reported either that they participated in an exercise program (such as gym, sports, jogging etc.) during their leisure time at least once a week, or that their occupation presupposes certain level of physical fatigue. The rest of the subjects were defined as physically inactive. Finally, we used data of self reported weight and height to calculate body mass index (BMI). Participants were classified as obese if BMI was ≥ 30 kg/m^2^.

### Statistical analysis

Continuous variables are presented as mean values ± standard deviation, while qualitative variables are presented as absolute and relative frequencies. Associations between categorical variables were tested by the use of contingency tables and the calculation of Pearson's chi-squared test. All comparisons among paired groups of sample in relation to normally distributed continuous variables were performed using the student t-test. Every reported *P*-value is based on two-sided tests and compared to a significance level of 5%. SPSS version 11.5 (Statistical Package for Social Sciences, SPSS Inc, U.S.A.) software was used for all the statistical calculations.

## Results

The characteristics (demographic characteristics, lifestyle habits and information from the medical history) of the study's participants in relation to gender are presented in Table 2. The mean age of men was slightly smaller than that of the women (43.7 ± 16.1 and 44.7 ± 16.0 years, respectively). Additionally, men were more educated and physically active, as well as more frequently current and former smokers.

**Table 2:** Characteristics of the study's participants (% by gender)

	Men (n = 2439)	Women (n = 2564)	P
Age (years)	43.7 ± 16.1	44.7 ± 16.0	0.025*
Education status ^a^			< 0.001*
Group I	710 (29.1 %)	997 (38.9 %)	
*Group II*	763 (31.3 %)	787 (30.7 %)	
*Group III*	966 (39.6 %)	779 (30.4 %)	
Physical status			< 0.001*
*Physically inactive*	1664 (67.8 %)	2110 (82.3 %)	
*Physically active*	784 (32.2 %)	454 (17.7 %)	
Smoking habits			< 0.001*
Never smoker	700 (28.7 %)	1381 (53.9 %)	
*Current smoker*	1176 (48.2 %)	901 (35.1 %)	
*Former smoker*	563 (23.1 %)	282 (11.0 %)	
BMI (Kg/m^2^) ^b^	26.3 ± 5.7	25.0 ± 5.3	< 0.001*
Obesity (BMI ≥ 30 Kg/m^2^)	322 (13.2 %)	345 (13.5 %)	0.703
Hypertension	325 (13.3 %)	455 (17.7 %)	< 0.001*
Hypercholesterolaemia	400 (16.4 %)	560 (21.8 %)	< 0.001*
Diabetes mellitus	152 (6.2 %)	146 (5.7 %)	0.422
Renal failure	23 (0.5 %)	39 (0.8 %)	0.065

^a ^The educational level of the participants was measured by the years of schooling (**Group I**: < 9 years, **Group II**: up to high school or technical colleges (10 – 14 years) and **Group III**: university

^b ^**BMI**: Body mass index.

P < 0.05 Statistical significant difference between men and women

Analysis of data relevant to the medical history showed statistically significant difference among men and women in relation to prevalence of hypercholesterolaemia and hypertension, but not to prevalence of obesity, diabetes mellitus and renal failure. In particular, 400 men (16.4%) and 560 women (21.8%) reported that they had hypercholesterolaemia, while 325 men (13.3%) and 455 women (17.7%) reported that they were hypertensive. Hypercholesterolaemic status was inversely associated with the prevalence of smoking and positively with the prevalence of the rest cardiovascular risk factors investigated (i.e. hypertension, diabetes mellitus, obesity, physical inactivity) in the overall sample. The prevalence's values of the aforementioned cardiovascular risk factors among normocholesterolaemic and hypercholesterolaemic subjects are presented in Table 3 (all *P*s < 0.001).

**Table 3:** Prevalence of cardiovascular risk factors in relation to hypercholesterolaemic status (in percentages)

	Normal (n = 4043)	Hypercholesterolaemic subjects (n = 960)	P
Hypertension	11.1 %	34.7 %	< 0.001
Diabetes Mellitus	4.2 %	13.3 %	< 0.001
Obesity	11.8 %	19.6 %	< 0.001
Smoking	43.2 %	34.3 %	< 0.001
Physical inactivity	73.2 %	84.1 %	< 0.001

The distribution of hypercholesterolaemic study's participants by age group, which is presented in Table 4, shows that the significant difference mentioned before between men and women in relation to hypercholesterolaemic status (women > men) is attributed to the large differences observed in older age groups (55 – 74 yr).

**Table 4:** Distribution of hypercholesterolaemic study's participants by age group

	Men		Women		P	Total	
Age	n		n			n	
18–24	333	2 (0.6 %)	317	7 (2.2 %)	*0.08*	650	9 (1.4 %)
25–34	518	24 (4.6 %)	510	25 (4.9%)	*0.84*	1028	49 (4.8 %)
35–44	474	66 (13.9 %)	496	48 (9.7%)	*0.04*	970	114 (11.8 %)
45–54	412	101 (24.5 %)	433	106 (24.5%)	*0.99*	845	207 (24.5 %)
55–64	358	110 (30.7 %)	405	177 (43.7%)	*< 0.001*	763	287 (37.6 %)
65–74	344	97 (28.2 %)	403	197 (48.9%)	*< 0.001*	747	294 (39.4 %)

Total	2439	400 (16.4 %)	2564	560 (21.8%)	*< 0.001*	5003	960 (19.2 %)

Difference was found between hypercholesterolaemic men and women relatively to the selected treatment of hypercholesterolaemia (P = 0.031). Table 5 present the respective distribution. Meanwhile, analysis of participants' family history of hypercholesterolaemia showed a positive association (P < 0.001) only between brothers (independently of gender) and not between parents and descendants. Specifically, only 2.1% of the normocholesterolaemic participants reported that they have a hypercolesterolaemic brother or a sister in contrast with 9.48% of hypercholesterolaemic participants who reported that they have a brother or a sister with the same health problem.

**Table 5:** Selected treatment of hypercholesterolaemia by study's participants (by gender, P = 0.031)

	**Men **(n = 400)	**Women **(n = 560)	**Total **(n = 960)
Drug treatment only	10.8 %	12.3 %	11.7 %
Dietary treatment only	48.5 %	51.4 %	50.2 %
Combined drug and dietary treatment	16.8 %	19.8 %	18.5 %
No treatment	24.0 %	16.4 %	19.6 %

Several associations were also detected between nutritional habits and hypercholesterolaemic status. The average weekly consumption (expressed in servings) of the investigated food groups by normocholesterolaemic and hypercholesterolaemic subjects is presented in Table 6. With the exception of some food groups or items i.e. chicken (P = 0.562), vegetables (P = 0.272) and legumes (P = 0.431), hypercholesterolaemic participants differ significantly when compared to normocholesterolaemics in relation to the consumption of the food groups examined. Specifically, statistic analysis of reported food consumption patterns showed that hypercholesterolaemics consume more frequently items from the groups of fish (P < 0.001), bread and cereals (P = 0.031), fruits and juices (P < 0.001) and low fat milk and yogurt (P < 0.001), while normocholesterolaemic participants have greater consumption of red meat (P < 0.001), pork (P < 0.001), egg (P < 0.001), pasta and rice (P < 0.001), potatoes (P < 0.001), full fat milk and yogurt (P < 0.001), yellow cheese (P < 0.001), white cheese (P < 0.001) and desserts or ice creams (P < 0.001).

**Table 6:** Food items consumed (in servings/week) in relation to hypercholesterolaemic status

	Normocholesterolaemic	Hypercholesterolaemic	P
Red meat	1.64 ± 1.28	1.44 ± 1.12	< 0.001*
Pork	1.27 ± 1.15	0.95 ± 0.99	< 0.001*
Poultry	1.42 ± 1.11	1.44 ± 0.96	0.562
Fish	1.31 ± 1.11	1.55 ± 1.15	< 0.001*
Egg	1.29 ± 1.63	0.69 ± 1.16	< 0.001*
Bread & Cereals	11.94 ± 8.76	12.61 ± 8.27	0.031*
Pasta & Rice	2.08 ± 1.50	1.69 ± 1.25	< 0.001*
Potatoes	2.25 ± 1.74	1.79 ± 1.43	< 0.001*
Vegetables	7.15 ± 3.81	7.30 ± 3.79	0.272
Fruits & Juices	7.45 ± 6.15	8.48 ± 6.92	< 0.001*
Milk & products Full Fat	3.77 ± 5.00	2.09 ± 3.88	< 0.001*
Milk & products Low Fat	2.27 ± 4.14	3.57 ± 4.83	< 0.001*
Cheese Yellow	2.26 ± 2.52	1.69 ± 2.24	< 0.001*
Cheese White	5.07 ± 3.29	4.47 ± 3.19	< 0.001*
Legumes	1.21 ± 0.94	1.24 ± 0.90	0.431
Dessert or Ice Cream	2.12 ± 2.68	1.59 ± 2.43	< 0.001*

P < statistical significant difference between normo- and hypercholesterolaemic participants

## Discussion

This cross – sectional study of about 5000 individuals from Greece investigated the prevalence of self-reported hypercholesterolemia and its relation to the nutritional habits of the participants. The main finding of this work was that about 1 out of 5 participants (overall population: 19.2%, men: 16.4%, women: 21.8%) reported of having high serum levels of total cholesterol. Meanwhile, the published epidemiological data regarding the current prevalence of hypercholesterolaemia in the Greek population are limited. In particular, the ATTICA study [17] is the only known, well organized and large – scale health survey that provided relevant data from an urban population. According to the aforementioned study, which was conducted to a sample from the province in which the capital of the country is located, the prevalence of clinical confirmed hypercholesterolaemia was 46.0% for men and 40.0% for women.

It is obvious that the respective figures of ATTICA study are around twofold of our estimations. Furthermore, our finding that hypercholesterolaemia is more prevalent in women than in men is also in contrast with the results of ATTICA study. The observed differences between the results of ATTICA study and our work can be possibly ascribed to the selected way of identifying hypercholesterolaemic participants in our study. Due to the fact that the participants self – reported whether they are hypercholesterolaemic (without confirmation via clinical examination), it is possible the prevalence's values to be underestimated as it is generally accepted that there is a large group of hypercholesterolaemic individuals unaware of their condition. Specifically, several studies [18-22] have reported that around half of those with hypercholesterolaemia are aware of their elevated cholesterol levels (the proportion varies from one-third to two-thirds across populations). Furthermore, according to the results from the WHO MONICA Project [16] (a multinational survey), the prevalence of awareness of hypercholesterolaemia was substantially higher in most populations among women. However, the fact that the prevalence of hypercholesterolaemia increases with age, which is a frequent finding of similar surveys, was also observed in the present work (Table 4).

The significant positive associations observed between the prevalence of hypercholesterolaemia and hypertension, diabetes mellitus, obesity and physical inactiity (Table 3) confirm the frequent finding from other studies [23-25], that hypercholesterolaemics usually, apart from high total cholesterol in serum, have to confront additional health problems, the coexistence of which enhances the total cardiovascular risk. However, the inverse association observed between the hypercholesterolamic status and the smoking habit is a "pleasant" finding since it possibly shows a trend among hypercholesterolaemic participants of avoiding this unhealthy habit in order to delay the progression of atherosclerosis. Therefore the above findings emphasize in the need for implementation of effective policies in Greece for the detection and control of hypercholesterolaemia.

In the present study, the expected higher prevalence of obesity (Table 3) in the subgroup of hypercholesterolaemic participants was confirmed, as well as the greater values of BMI in the same subgroup (hypercholesterolaemics: 27.21 ± 6.47 kg/m^2^, normocholesterolaemics: 25.21 ± 5.25 kg/m^2^, p < 0.001). This finding shows that although hypercholesterolaemic participants are aware of their hypercholesterolaemic status, they have not managed yet to approach an ideal body weight. However, analysis of the reported food consumption patterns suggests that they might have started adopting some more healthy nutritional behaviour compared to normocholesterolaemic ones. Specifically, as it was mentioned in the results section, consumption of fish, fruits and juices, cereals, and low fat milk and yogurt was found to be higher among hypercholesterolaemic subjects while the opposite was observed for food items as red meat, pork, egg, full fat dairy products and desserts (Table 6). This finding possibly reveals a first level of compliance of the Greek hypercholesterolaemic subpopulation to the respective dietary guidelines. The considerable differences observed among the two subpopulations in relation to the consumption of eggs and full fat dairy products (about twofold) support this hypothesis, since avoidance of the over-consumption of the specific food items, due to their high content of saturated fat, is a very common guideline to people with hypercholesterolaemia. Furthermore, the majority of the hypercholesterolaemic participants (65.3% of men and 71.2% of women) reported (Table 5) implementation of dietary treatment (combined or not with drug treatment), which possibly explains the observed trend towards a more healthy food consumption pattern. However, the reported food consumptions are quite different from the recommended ones, according to the beneficial dietary pattern of Mediterranean Diet [10,26,27] which among others highlights the importance of adequate consumption of vegetables and legumes.

### Limitations

This study as a cross-sectional one cannot establish causal relations but only generate hypothesis that could be evaluated by future prospective randomized trials. Additionally, the applied method of self – reporting hypercholesterolaemia neither is able to provide data relevant to the level of hypercholesterolaemia's awareness (thus the evaluated prevalence of hypercholesterolaemia may be underestimated), nor can be as accurate as the biochemical tests. However, Natarajan et al. [28], who investigated the validity of self-reported hypercholesterolaemia in the U.S. adult population, suggested that this method may be used for surveillance of hypercholesterolaemia trends in the absence of measured cholesterol levels, but caution should be given when interpreting the results due to low sensitivity of the method. Another methodological limitation of the present study is that analysis of semi-quantitative food frequency questionnaires cannot provide accurate data in terms of specific nutrients, so conclusions should be expressed only in relation to food groups or items tested.

## Conclusion

According to the presented data, hypercholesterolaemia affects a large part of the Greek population. Possibly the adoption of "westernized" dietary patterns by Greeks during last decades, played a strong role for this phenomenon. Since, effective treatment of hypercholesterolaemia is an important element of strategies to prevent cardiovascular disease, we suggest that periodic cholesterol measurement is considered essential and must be followed by active and effective interventions.

## References

[B1] Kromhout D (2001). Epidemiology of cardiovascular diseases in Europe. Public Health Nutr.

[B2] Gotto AMJ, Pearson TA, Criqui MH, Luepker RV, Oberman A, Wilson M (1994). Lipid and lipoproteins disorders. Primer in Preventive Cardiology.

[B3] Ginsberg HN (1994). Lipoprotein metabolism and its relationship to atherosclerosis. Med Clin North Am.

[B4] Wilson PW, Abbott RD, Castelli WP (1988). High density lipoprotein cholesterol and mortality. The Framingham Heart Study. Arteriosclerosis.

[B5] (2001). Executive Summary of The Third Report of The National Cholesterol Education Program (NCEP) Expert Panel on Detection, Evaluation, And Treatment of High Blood Cholesterol In Adults (Adult Treatment Panel III). Jama.

[B6] Gran B (1995). Major differences in cardiovascular risk indicators by educational status. Results from a population based screening program. Scand J Soc Med.

[B7] http://www.who.int/cardiovascular_diseases/resources/atlas/en/.

[B8] Denke MA (1995). Cholesterol-lowering diets. A review of the evidence. Arch Intern Med.

[B9] Trichopoulou A, Kouris-Blazos A, Wahlqvist ML, Gnardellis C, Lagiou P, Polychronopoulos E, Vassilakou T, Lipworth L, Trichopoulos D (1995). Diet and overall survival in elderly people. Bmj.

[B10] de Lorgeril M, Salen P, Martin JL, Monjaud I, Delaye J, Mamelle N (1999). Mediterranean diet, traditional risk factors, and the rate of cardiovascular complications after myocardial infarction: final report of the Lyon Diet Heart Study. Circulation.

[B11] Pitsavos C, Panagiotakos DB, Chrysohoou C, Skoumas J, Papaioannou I, Stefanadis C, Toutouzas PK (2002). The effect of Mediterranean diet on the risk of the development of acute coronary syndromes in hypercholesterolemic people: a case-control study (CARDIO2000). Coron Artery Dis.

[B12] Trichopoulou A, Costacou T, Bamia C, Trichopoulos D (2003). Adherence to a Mediterranean diet and survival in a Greek population. N Engl J Med.

[B13] Salonen JT, Puska P, Kottke TE, Tuomilehto J (1981). Changes in smoking, serum cholesterol and blood pressure levels during a community-based cardiovascular disease prevention program--the North Karelia Project. Am J Epidemiol.

[B14] (1984). The Lipid Research Clinics Coronary Primary Prevention Trial results. I. Reduction in incidence of coronary heart disease. Jama.

[B15] Blankenhorn DH, Nessim SA, Johnson RL, Sanmarco ME, Azen SP, Cashin-Hemphill L (1987). Beneficial effects of combined colestipol-niacin therapy on coronary atherosclerosis and coronary venous bypass grafts. Jama.

[B16] Tolonen H, Keil U, Ferrario M, Evans A (2005). Prevalence, awareness and treatment of hypercholesterolaemia in 32 populations: results from the WHO MONICA Project. Int J Epidemiol.

[B17] Panagiotakos DB, Pitsavos C, Chrysohoou C, Skoumas J, Stefanadis C (2004). Status and management of blood lipids in Greek adults and their relation to socio-demographic, lifestyle and dietary factors: the ATTICA Study. Blood lipids distribution in Greece. Atherosclerosis.

[B18] Gnasso A, Calindro MC, Carallo C, De Novara G, Ferraro M, Gorgone G, Irace C, Romeo P, Siclari D, Spagnuolo V, Talarico R, Mattioli PL, Pujia A (1997). Awareness, treatment and control of hyperlipidaemia, hypertension and diabetes mellitus in a selected population of southern Italy. Eur J Epidemiol.

[B19] Nieto FJ, Alonso J, Chambless LE, Zhong M, Ceraso M, Romm FJ, Cooper L, Folsom AR, Szklo M (1995). Population awareness and control of hypertension and hypercholesterolemia. The Atherosclerosis Risk in Communities study. Arch Intern Med.

[B20] Salomaa V, Korhonen HJ, Tuomilehto J, Vartiainen E, Pietinen P, Kartovaara L, Gref CG, Nissinen A, Puska P (1990). Serum cholesterol distribution, measurement frequency and cholesterol awareness in three geographical areas of Finland. Eur Heart J.

[B21] Marques-Vidal P, Arveiler D, Evans A, Amouyel P, Ferrieres J, Luc G, Ducimetiere P (2002). Awareness, treatment and control of hyperlipidaemia in middle-aged men in France and northern ireland in 1991-1993: the PRIME study. Prospective epidemiological study of myocardial infarction. Acta Cardiol.

[B22] Burke GL, Sprafka JM, Folsom AR, Hahn LP, Luepker RV, Blackburn H (1991). Trends in serum cholesterol levels from 1980 to 1987. The Minnesota Heart Survey. N Engl J Med.

[B23] Foucan L, Bangou-Bredent J, Ekouevi DK, Deloumeaux J, Roset JE, Kangambega P (2001). Hypertension and combinations of cardiovascular risk factors. An epidemiologic case-control study in an adult population in Guadeloupe (FWI). Eur J Epidemiol.

[B24] Rudnichi A, Safar M, Asmar R, Guize L, Benetos A (1998). Prevalence of cardiovascular risk factors in a French population. J Hypertens Suppl.

[B25] Castelli WP, Anderson K (1986). A population at risk. Prevalence of high cholesterol levels in hypertensive patients in the Framingham Study. Am J Med.

[B26] Willett WC, Sacks F, Trichopoulou A, Drescher G, Ferro-Luzzi A, Helsing E, Trichopoulos D (1995). Mediterranean diet pyramid: a cultural model for healthy eating. Am J Clin Nutr.

[B27] Kris-Etherton P, Eckel RH, Howard BV, St Jeor S, Bazzarre TL (2001). AHA Science Advisory: Lyon Diet Heart Study. Benefits of a Mediterranean-style, National Cholesterol Education Program/American Heart Association Step I Dietary Pattern on Cardiovascular Disease. Circulation.

[B28] Natarajan S, Lipsitz SR, Nietert PJ (2002). Self-report of high cholesterol: determinants of validity in U.S. adults. Am J Prev Med.

